# Progression of Neuropsychiatric Symptoms over Time in an Incident Parkinson’s Disease Cohort (ICICLE-PD)

**DOI:** 10.3390/brainsci10020078

**Published:** 2020-02-02

**Authors:** J. K. Dlay, G. W. Duncan, T. K. Khoo, C. H. Williams-Gray, D. P. Breen, R. A. Barker, D. J. Burn, R. A. Lawson, A. J. Yarnall

**Affiliations:** 1 Institute of Neuroscience, Newcastle University, Newcastle upon Tyne NE4 5PL, UK; JKD462@student.bham.ac.uk (J.K.D.); gordon.duncan@ed.ac.uk (G.W.D.); rachael.lawson@ncl.ac.uk (R.A.L.); 2 Centre for Clinical Brain Sciences, University of Edinburgh, Edinburgh EH16 4SB, UK; 3 School of Medicine and Menzies Health Institute Queensland, Griffith University, Gold Coast 4222, Australia; t.khoo@griffith.edu.au; 4 School of Medicine, University of Wollongong, New South Wales 2522, Australia; 5 John van Geest Centre for Brain Repair, University of Cambridge, Cambridge CB2 0PY, UK; chm27@cam.ac.uk (C.H.W.-G.); rab46@cam.ac.uk (R.A.B.); 6 Anne Rowling Regenerative Neurology Clinic, University of Edinburgh, Edinburgh EH16 4SB, UK; 7 Usher Institute of Population Health Sciences and Informatics, University of Edinburgh, Edinburgh EH16 4UX, UK; 8 Faculty of Medical Sciences, Newcastle University & Newcastle upon Tyne Hospitals NHS Foundation Trust, Newcastle upon Tyne NE2 4HH, UK; david.burn@newcastle.ac.uk; 9 Newcastle upon Tyne Hospitals NHS Foundation Trust, Newcastle upon Tyne NE7 7DN, UK

**Keywords:** neuropsychiatric symptoms, Parkinson’s disease, quality of life, non-motor

## Abstract

Background: Cross-sectional studies have identified that the prevalence of neuropsychiatric symptoms (NPS) in Parkinson’s disease (PD) ranges from 70–89%. However, there are few longitudinal studies determining the impact of NPS on quality of life (QoL) in PD patients and their caregivers. We seek to determine the progression of NPS in early PD. Methods: Newly diagnosed idiopathic PD cases (*n* = 212) and age-matched controls (*n* = 99) were recruited into a longitudinal study. NPS were assessed using the Neuropsychiatric Inventory with Caregiver Distress scale (NPI-D). Further neuropsychological and clinical assessments were completed by participants, with reassessment at 18 and 36 months. Linear mixed-effects modelling determined factors associated with NPI-D and QoL over 36 months. Results: Depression, anxiety, apathy and hallucinations were more frequent in PD than controls at all time points (*p* < 0.05). Higher motor severity at baseline was associated with worsening NPI-D scores over time (β = 0.1, *p* < 0.05), but not cognition. A higher NPI total score was associated with poorer QoL at any time point (β = 0.3, *p* < 0.001), but not changed in QoL scores. Conclusion: NPS are significantly associated with poorer QoL, even in early PD. Screening for NPS from diagnosis may allow efficient delivery of better support and treatment to patients and their families.

## 1. Introduction

Neuropsychiatric symptoms (NPS) are common in Parkinson’s disease (PD), with some studies demonstrating a prevalence of 70–89% [1,2,3]. They can have a major impact on the lives of patients and their families by contributing to morbidity, risk of institutionalisation [4], increased healthcare costs [5] and carer burden [6]. A recent study showed that NPS are missed in roughly half of consultations [7], which may delay symptom recognition and management [8].

Depression is one of the most common NPS, occurring in almost 60% of PD patients [1]. Anxiety [9], apathy and irritability are also common [3]. To date, there is a paucity of evidence on the burden of NPS in PD and their progression over time, with the majority of research performed in cross-sectional studies [1,3,10,11]. NPS are usually associated with the later stages of PD when they commonly occur alongside a PD dementia (PDD); however, greater understanding of NPS in the earlier stages of disease would be beneficial. We thus sought to identify the frequency and progression of NPS over time in newly diagnosed PD cases compared to age-matched controls and to determine predictors of NPS severity and QoL.

## 2. Materials and Methods

### 2.1. Participants

This study was conducted as part of the Incidence of Cognitive Impairment in Cohorts with Longitudinal Evaluation—Parkinson’s disease (ICICLE-PD) study, which originally recruited PD participants between June 2009 and December 2011 from the Newcastle upon Tyne region and Cambridgeshire [12]. Participants were diagnosed by a movement disorder specialist and fulfilled UK PD Brain Bank criteria [13]. Healthy controls recruited from the community in the North East were matched with regards to age and sex. Exclusion criteria were Mini Mental State Examination (MMSE) score < 24 or a diagnosis of dementia [14], atypical parkinsonian syndrome, vascular parkinsonism, drug-induced parkinsonism, prevalent cases, and those without the capacity to consent.

This study was approved by the Newcastle and North Tyneside Research Ethics Committee and performed in line with the Declaration of Helsinki. Participants provided written informed consent.

### 2.2. Assessments

Demographic data were collected, including age, sex, co-morbidities, medication, along with years of education. Depression was assessed using the Geriatric Depression Scale-15 (GDS-15) [15]. Global cognitive function was assessed using the Montreal Cognitive Assessment (MoCA) [16]. Disease severity was assessed using the Movement Disorder Society revised Unified Parkinson’s Disease Rating Scale (MDS-UPDRS) parts II and III [17] and Hoehn and Yahr [18]. The Parkinson’s Disease Questionnaire-39 (PDQ-39) [19] summary index (SI) was used as a measure of global QoL. This validated tool comprises scores ranging from 0 (best QoL) to 100 (worst possible QoL score). PD participants were assessed whilst “on” PD medication and their levodopa equivalent daily dose (LEDD) was calculated [20].

Informants (partners, adult family members or friends of the participants) completed the Neuropsychiatric Inventory Caregiver Distress (NPI-D) scale [21], a validated measure of neuropsychiatric symptoms and carers’ level of distress. The NPI-D assesses the frequency and severity of 12 neuropsychological symptoms and a composite score is obtained for each item, in addition to a score for caregiver levels of distress. NPI total score ranges from 0–144, while caregiver distress ranges from 0–60 (with higher scores indicating a greater disturbance of behaviour or levels of distress, respectively). Participants were reassessed at 18 and 36 months.

### 2.3. Statistical Analyses

Statistical analyses were undertaken using SPSS (Version 24; SPSS, Inc., Chicago, IL, USA). The data were tested for normality using the Kolmogorov–Smirnov test and visualised using histograms and boxplots. Mann–Whitney *U*-tests and independent *t*-tests were performed to compare differences between the two groups as appropriate. Chi-squared tests were performed for categorical data; where a sample size was less than five, a Fisher’s exact test was used. McNemar’s test was used to assess changes in NPS frequency over time. A *p*-value of < 0.05 was considered significant in all tests undertaken. 

R software (Version 3.4.0; R Foundation for Statistical Computing, Vienna, Austria) and lme4 were used to perform a linear mixed-effects analysis of the relationship between clinical measures on the NPS over 36 months. Due to the longitudinal nature of this study, there were some missing data. This form of multilevel modelling is suitable for longitudinal data analysis due to its ability to handle missing data [22], as it does not remove participant data list-wise. A random intercept model was used, where the intercept varied at the participant and time level. Change in NPI total score over 36 months was first determined, with separate models for PDs and controls. Baseline age, sex, years of education and LEDD score were entered into the model as fixed effects, as well as interactions of time age (age × LEDD) and LEDD (Time × LEDD). Depression (GDS-15) was not included in the model due to this domain being measured as part of the NPI-D scoring and was therefore not an independent variable. A reduced model was produced by excluding non-significant predictors to which measures of disease severity (MDS-UPDRS III) and cognition (MoCA) were then added. This method was repeated to determine the predictors of carer distress. Fit of the models was assessed by likelihood ratio tests.

Linear mixed-effects models were used to determine whether the NPI total score was a significant, independent predictor of QoL over 36 months. A random intercept model was used, where the intercept varied at the participant and time level. Age, sex, years of education, LEDD, MoCA and MDS-UPDRS III score were entered into the model as fixed effects, as well as interactions of time with cognition (MoCA × Time), motor severity (Time × MDS-UPDRS III) and LEDD (Time × LEDD). As previously, depression (GDS-15) was not included in the model due to this domain also being measured as part of the NPI. A reduced model was produced by excluding non-significant predictors to which NPI total and interactions with time were then added. The Benjamini–Hochberg procedure was used to correct for multiple comparisons in all analyses.

## 3. Results

**Figure 1:**
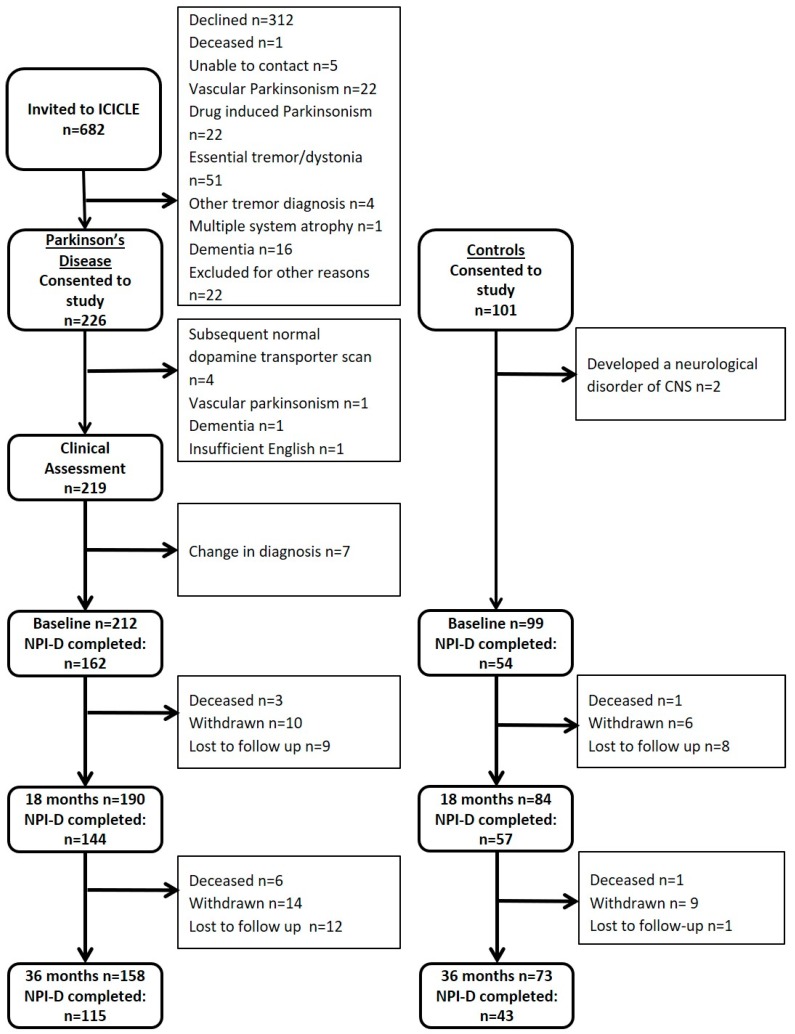
Flowchart outlining participants.

At baseline, the average time from disease diagnosis in the PD group was 6.1 months. A percentage of PD participants (76.4%; *n* = 162) had informants who completed the NPI-D at baseline and 72% (*n* = 115) completed it at 36 months (Figure 1). In controls, 55% (*n* = 54) and 59% (*n* = 43) of the informants completed the NPI-D at baseline and 36 months, respectively. There were no significant differences in baseline demographics, NPI total score, caregiver distress score, QoL or global cognition between those who were and those who were not included in this analysis (*p* > 0.05 for all; data not shown).

Comparison of baseline characteristics for PD vs. controls (Table 1) showed that they were well matched for age, gender and level of education (*p* > 0.05 for all). Consistently at each time point, PD participants scored significantly lower for global cognition, and higher for depression, NPI total score and NPI caregiver distress score compared to controls (*p* < 0.01 for all).

### 3.1. Comparisons of NPS in PD VS. Controls

**Figure 2:**
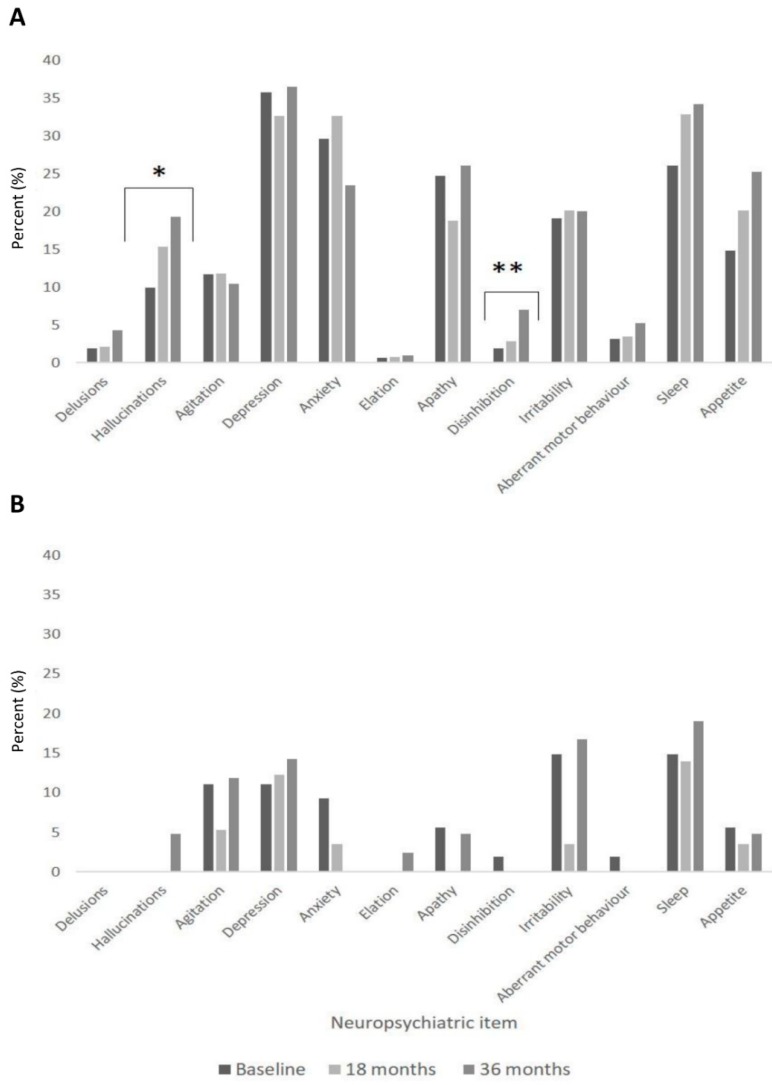
Progression of neuropsychiatric symptoms over time in PD (**A**) vs. controls (**B**).

The frequency of NPS in PD and control participants was examined. Depression was the most common symptom experienced by the PD cohort (35.8%, 32.6% and 36.5% at baseline, 18 and 36 months, respectively). Anxiety, apathy and hallucinations were also reported frequently in PD participants (Figure 2A). In controls (Figure 2B), the most common symptom experienced at each time point was sleep disturbances (14.8% at baseline), but there were no significant changes for NPS over time (*p* > 0.05 for all). Comparison of NPS between baseline and 36-month assessments established that in PD participants, hallucinations (*p* = 0.002) and disinhibition (*p* = 0.031) significantly increased in frequency over time (Figure 2A), but no other significant differences were found (*p* > 0.05 for all). There were no significant changes in NPS frequency over time in controls.

PD participants reported significantly higher frequencies of hallucinations (9.9% vs. 0%), depression (35.8% vs. 11.1%), anxiety (29.6% vs. 9.3%) and apathy (24.7% vs. 5.6%) compared to controls (*p* < 0.05 for all) at all time points. At 18 months, irritability and sleep disturbances were higher in PD compared to controls (20.1% vs. 3.5% and 32.9% vs. 14.0%, respectively, *p* < 0.01). Appetite changes were significantly higher in PD participants compared to controls at 18 months (20.1% vs. 3.5%) and 36 months (25.2% vs. 4.8%, *p* < 0.01 for both).

### 3.2. Factors Associated with NPS

Linear mixed-effects modelling was performed to determine baseline predictors of change in NPI total score and carer distress total in PD and control participants over 36 months. No significant predictors of NPI over time were identified using a data-driven approach, therefore age, sex and time were used in all models as covariates (Table 2). In PD participants, increased baseline motor severity was associated with increasing NPI total score over time (β = 0.1, *p* = 0.044), adjusting for baseline age and sex; log-likelihood ratio comparing the fit of the basic model showed that including the MDS-UPDRS III over time significantly improved the model (*χ*^2^ = 6.8, *p* = 0.009). No significant associations were found between NPI total score and MoCA scores for PD or control participants. 

Modelling carer distress scores found that increased baseline motor severity was significantly associated with increasing NPI carer distress score over time (β = 0.1, *p* = 0.002, Table 2) and significantly improved the fit of the model (*χ*^2^ = 5.0, *p* = 0.083). Higher baseline MoCA score was significantly associated with lower NPI carer distress total (β = −0.3, *p* = 0.007), but not changed over time. No significant associations were found in control participants.

### 3.3. Factors Associated with QoL

To determine whether NPS were significantly associated with QoL over time, predictors of change in PDQ-39 scores over 36 months were determined. After excluding non-significant predictors, being female, lower years of education, younger age, increased dopaminergic medication (LEDD), increased motor severity (MDS-UPDRS III) and decline in MoCA scores were significantly associated with declining QoL (*p* < 0.05 for all, Table 3); only age, motor severity and declining cognition remained significant after Benjamini–Hochberg correction for multiple comparisons. 

NPI total and NPI carer distress scores plus interactions with time were separately added to the basic model. Increased NPI total score (β = 0.3, *p* < 0.001) but not its interaction with time (*p* > 0.05) was associated with poorer QoL, suggesting the cross-sectional NPS burden was associated with poorer QoL scores at any time point, but not associated with declining QoL. Similarly, increased NPI carer distress score but not its interaction with time was associated with poorer QoL. This suggests that cross-sectional carer distress due to NPS was associated with poorer QoL scores at any time point, but not associated with declining QoL. These associations remained significant after Benjamini–Hochberg correction.

## 4. Discussion

To our knowledge, this is the first longitudinal study to explore the natural history of NPS over time in newly diagnosed PD. We demonstrated that such patients experienced increased NPS burden and caregiver distress compared to controls over the 36-month study period. In PD, increased NPS was associated with worsening motor severity over 36 months and contributed to poorer QoL, but was not associated with changes in cognition. Depression was the most common symptom experienced in newly diagnosed PD patients, with anxiety, apathy and hallucinations also frequently reported. Hallucinations and disinhibition significantly increased over time. 

Similar findings have been reported by Weintraub et al. [11], where depression, apathy and anxiety were also higher in PD patients compared to controls in a larger sample of newly diagnosed PD subjects (*n* = 423) and controls (*n* = 196). We found hallucinations were also common and increased in frequency along with agitation over time. This is consistent with findings reported by Williams et al. [23] and Holroyd et al. [24], who found that up to half of participants experienced visual hallucinations. This is an important finding since hallucinations are associated with greater severity of disease [25] and the development of PDD [26]. This can have a negative impact on carers and increases risk of care home placement [27].

Consistent with our findings, carer distress in NPS has been reportedly higher in PD compared to controls in previous research [28]. Almost half of the carers of newly diagnosed PD participants reported distress using the NPI-D assessment compared to 16% of controls, with over 25% of PD carers experiencing moderate to severe distress at baseline. In the present study, we have shown carers’ distress scores of PD participants were almost four times that of control informants even at diagnosis. This may be explained by increasing responsibilities and strain on the relationship affecting carers’ QoL [1,28,29,30].

Over the 36-month study period, increased baseline motor severity was associated with an increased NPI total score and increased carer distress. This is in keeping with current knowledge that NPS are more frequently seen in the later stages of PD and hence more advanced motor stages of the condition [1]. Contrary to our hypotheses, the MoCA was not significantly associated with the NPI total score. Previous studies have shown associations between global cognition and NPS burden. Aarsland et al. [1] showed that patients with a lower MMSE were likely to have greater NPS, supported by further research by Leroi et al. [31]. Compared to previous studies, participants in our cohort had less advanced disease; participants’ had a mean PD duration of 6 months at baseline and were largely Hoehn and Yahr stage 1 or 2. 

Similar to previous prevalent cohort studies [28,29,30,31,32,33], we found that NPS were a significant contributor to poorer quality of life in PD participants. The significance of our work is that this is evident even in newly diagnosed participants. Previous research has shown depression, hallucinations and anxiety are associated with poorer QoL [34]. However, further research is needed to fully understand and appreciate the role of other NPS and their effect on the QoL of patients [35]. It has been reported that these NPS are under-recognised [36] and thus undertreated; our results highlight the importance of screening for these symptoms in the early stages of disease which consequently may lead to improvements in patient’s QoL and reduce caregiver distress [28].

The strengths of this study are the use of data from a large representative community-based population of newly diagnosed patients with idiopathic PD. Additionally, the use of a control group allowed for comparisons with normal ageing. Our results draw attention to the significant differences between the two groups; this is an important finding as it highlights the increased prevalence of NPS in PD compared to the healthy population. This study replicates and extends the findings of previous cross-sectional studies [1,3,11]. The progression of NPS over time in incident PD participants compared to controls in the current literature has not been studied longitudinally. 

The limitations of our study include the fact that the participants were not drug naïve; therefore it was not possible to determine whether medication had an influence on NPS, although our findings represent patients seen in normal routine clinical practice. The NPI-D tool [21] is also subject to inter-rater variability, although we attempted to minimise this by using a trained researcher to ask the informant about their NPS. Moreover, participants at baseline had an average duration of disease of 6 months and therefore some NPS may be mild and hence underreported. We note that GDS-15, NPI and NPI-D scores were relatively low across the cohort at baseline, and thus potentially our patients were not very burdened by these symptoms. Due to the longitudinal nature of the study, participant drop-out was inevitable and it is possible that those who withdrew may have been experiencing greater NPS and so we may have underestimated their frequency. We acknowledge that, as in any longitudinal study, there was a degree of attrition, and although there was a lack of baseline differences in NPS between those who did and did not drop out, this may not necessarily reflect the trajectory and rate of decline over time. This may be particularly true for tests with a high ceiling or floor effect, such as the MoCA and GDS-15, respectively, which thus may have introduced bias. However, we used linear mixed-effects modelling in our analysis, which has the advantage that it is able to handle missing data and so does not remove participant data list-wise, which helps to mediate some of this bias. 

## 5. Conclusions

Neuropsychiatric symptoms and caregiver distress were greater in newly diagnosed PD participants compared to controls and were significantly associated with poorer QoL. These findings can be utilised in clinical practice as they highlight the importance of screening for NPS early in the course of the disease. Results from this study identified the most prevalent NPS in early PD, which were depression, anxiety, apathy and hallucinations. A major unmet research need is to find effective treatments for these symptoms, thus helping to alleviate distress amongst patients and their carers.

## Figures and Tables

**Table 1:** Comparison of demographic and clinical data in the Neuropsychiatric Inventory with Caregiver Distress scale (NPI-D) completers.

	Baseline				18 Months				36 Months			
	PD *n* = 162	Control *n* = 54	T/z/*χ*^2^	*p*-value	PD *n* = 144	Control *n* = 57	T/z/*χ*^2^	*p*-value	PD *n* = 115	Control *n* = 42	T/z/*χ*^2^	*p*-value
**Age (years)**	66.2 (10.1)	67.5 (7.5)	−1.0	0.335 †	68.3 (9.4)	68.6 (7.9)	−0.2	0.830 †	69.3 (9.5)	70.6 (6.4)	−1.0	0.313 †
**Sex (Male) *n* (%)**	104 (64.2)	30 (55.6)	1.3	0.257 *	97 (67.4)	30 (52.6)	3.8	0.051 *	79 (68.7)	23 (54.8)	2.6	0.105 *
**Education (years)**	12.5 (3.5)	13.0 (3.3)	−1.1	0.261	12.8 (3.5)	13.1 (3.2)	−0.9	0.373	12.7 (3.3)	12.8 (3.2)	−0.3	0.801
**Disease duration (months)**	6.1 (5.4)	-	-	-	24.1 (23.4)	-	-	-	42.1 (41.4)	-	-	-
**GDS-15**	3.1 (2.7)	1.0 (1.6)	−6.0	**<0.001**	2.9 (2.7)	1.2 (2.0)	−5.2	**<0.001**	3.1 (2.6)	1.2 (2.0)	−5.0	**<0.001**
**MoCA ^a^**	25.3 (3.4)	27.2 (2.2)	−3.6	**<0.001**	26.1 (3.7)	27.6 (2.7)	−2.9	**0.004**	25.6 (3.7)	27.4 (3.0)	−3.0	**0.002**
**NPI total score**	6.7 (9.8)	1.9 (4.3)	−4.5	**<0.001**	6.4 (9.5)	1.3 (3.3)	−5.4	**<0.001**	7.2 (9.8)	3.3 (8.3)	−4.3	**<0.001**
**NPI-Caregiver distress score**	3.3 (4.8)	0.9 (2.2)	−4.4	**<0.001**	2.7 (4.0)	0.6 (1.9)	−4.7	**<0.001**	3.8 (5.3)	1.5 (4.0)	−4.0	**<0.001**
**PDQ-39**	19.0 (14.3)	-	-	-	21.1 (16.3)	-	-	-	22.3 (17.2)	-	-	-
**MDS-UPDRS-II**	10.2 (5.9)	-	-	-	11.8 (6.0)	-	-	-	14.5 (7.6)	-	-	-
**MDS-UPDRS-III**	27.7 (12.3)	-	-	-	33.3 (12.1)	-	-	-	35.2 (15.0)	-	-	-
**Hoehn and Yahr**	1.9 (0.7)	-	-	-	2.2 (0.5)	-	-	-	2.1 (0.6)	-	-	-
**LEDD (mg/day)**	190.4 (159.9)	-	-	-	413.8 (214.3)	-	-	-	518.2 (273.5)	-	-	-
**PDD *n* (%)**	0 (0)	-	-	-	8 (5.6)	-	-	-	14 (12.2)	-	-	-

All data presented are means (standard deviations) except where indicated. All data are non-parametric and test statistics are *z*-scores using Mann–Whitney *U*-test except where indicated. † = independent *t*-test, * = chi-squared. Significant values indicated in bold. Abbreviations: GDS-15 = Geriatric Depression Scale-15, MoCA = Montreal Cognitive Assessment, NPI-D = Neuropsychiatric Inventory with Carer Distress Scale, PDQ-39 = Parkinson’s Disease Questionnaire, MDS-UPDRS = Movement Disorder Society Unified Parkinson’s Disease Rating Scale, LEDD = levodopa equivalent daily dose, PDD = PD dementia. ^a^ At baseline, *n* = 23 did not complete MoCA.

**Table 2:** Baseline predictors of change in NPI total and NPI carer distress total using mixed-effects modelling.

	PD				Control			
	β	SE	*t*-value	*p*-value	β	SE	*t*-value	*p*-value
**NPI total**								
*Basic model*								
Sex (Male)	1.2	1.3	1.0	0.329	−0.8	1.0	−0.847	0.399
Age	−0.1	0.1	−1.6	0.105	0.1	0.1	0.940	0.350
Time	0.4	0.4	0.9	0.390	0.6	0.5	1.193	0.235
*Basic model + MDS-UPDRS III*								
MDS-UPDRS III	0.0	0.1	0.6	0.563				
MDS-UPDRS III × Time	0.1	0.0	2.0	**0.044**				
*Basic model + MoCA*								
MoCA	−0.4	0.2	−1.8	0.075	0.0	147.6	0.1	0.900
MoCA × Time	−0.1	0.1	−0.7	0.457	−0.5	126.3	−1.9	0.057
**NPI carer distress total**								
*Basic model*								
Sex (Male)	0.8	0.6	1.3	0.206	0.1	0.5	0.124	0.901
Age	0.0	0.0	−0.3	0.791	0.0	0.0	0.525	0.601
Time	0.3	0.3	1.0	0.404	0.3	0.3	1.119	0.265
*Basic model + MDS-UPDRS III*								
MDS-UPDRS III	0.0	0.0	0.1	0.957				
MDS-UPDRS III × Time	0.1	0.0	3.1	**0.002**				
*Basic model + MoCA*								
MoCA	−0.3	0.1	−2.7	**0.007**	0.0	147.8	−0.2	0.865
MoCA × Time	0.0	0.1	−0.8	0.432	−0.2	127.2	−1.9	0.065

Basic model includes age, sex and time as covariates. Figures highlighted in bold indicate significant results after Benjamini–Hochberg procedure. Abbreviations: MoCA = Montreal Cognitive Assessment, MDS-UPDRS III = Movement Disorder Society Unified Parkinson’s Disease Rating Scale part III.

**Table 3:** Predictors of change in quality of life (PDQ-39) using mixed-effects modelling.

Basic Model	β	SE	*t* Value	*p*-Value
**Basic model**				
Sex (Female)	−3.4	1.7	−2.1	0.041
Education (Years)	−0.6	0.2	−2.5	0.012
Age	−0.3	0.1	−3.9	**<0.001**
LEDD	0.0	0.0	3.1	0.002
MDS-UPDRS III	0.4	0.0	8.3	**<0.001**
Time (Assessment)	12.5	3.4	3.7	**<0.001**
MoCA	−0.1	0.2	−0.4	0.654
MoCA × Time	−0.5	0.1	−3.8	**<0.001**
**Basic model + NPI Total**				
NPI Total	0.3	0.1	3.9	**<0.001**
NPI Total × Time	0.0	0.1	0.6	0.551

Basic model includes age, sex, years of education, MoCA, LEDD, MDS-UPDRS Part III, MoCA over time as covariates. Figures highlighted in bold indicate significant results after Benjamini–Hochberg procedure. Abbreviations: PDQ-39 = Parkinson’s Disease Questionnaire, MoCA = Montreal Cognitive Assessment, LEDD = levodopa equivalent daily dose, MDS-UPDRS III = Movement Disorder Society Unified Parkinson’s Disease Rating Scale part III, NPI = Neuropsychiatric Inventory.
